# Supplementary material for: Biocompatibility of Root Canal Sealers: A Systematic Review of In Vitro and In Vivo Studies
Source: Materials (Basel). 2019 Dec 9;12(24):4113. doi: 10.3390/ma12244113 (PMC6947586; doi:10.3390/ma12244113)
Supplement: Supplementary file 1 [file materials-12-04113-s001.pdf]

materials

Supplementary Materials

Biocompatibility of Root Canal Sealers: A Systematic Review of In Vitro and In Vivo Studies

Diogo Afonso Fonseca [1,*], Anabela Baptista Paula [2], Carlos Miguel Marto [2,3], Ana Coelho [2], Siri Paulo [4], José Pedro Martinho [4], Eunice Carrilho [2] and Manuel Marques Ferreira [4]

[1] Institute of Endodontics, Coimbra Institute for Clinical and Biomedical Research (iCBR), CIBB Center for Innovative Biomedicine and Biotechnology, Faculty of Medicine, University of Coimbra; 3000-075 Coimbra, Portugal

[2] Institute of Integrated Clinical Practice, Coimbra Institute for Clinical and Biomedical Research (iCBR), CIBB Center for Innovative Biomedicine and Biotechnology, CIMAGO – Center of Investigation on Environment, Genetics and Oncobiology, CNC.IBILI, Faculty of Medicine, University of Coimbra; 3000-075 Coimbra, Portugal; anabelabppaula@sapo.pt (A.B.P.), mig-marto@hotmail.com (C.M.M.), anasofiacoelho@gmail.com (A.C.), eunicecarrilho@gmail.com (E.C.)

[3] Institute of Experimental Pathology, Faculty of Medicine, University of Coimbra; 3000-075 Coimbra, Portugal

[4] Institute of Endodontics, Coimbra Institute for Clinical and Biomedical Research (iCBR), CIBB Center for Innovative Biomedicine and Biotechnology, CIMAGO – Center of Investigation on Environment, Genetics and Oncobiology, CNC.IBILI, Faculty of Medicine, University of Coimbra; 3000-075 Coimbra, Portugal; sirivpaulo@gmail.com (S.P.), josepedromartinho@gmail.com (J.P.M.), m.mferreira@netcabo.pt (M.M.F.)

* Correspondence: diogo.andre.fonseca@gmail.com; Tel.: +351-239-249-151

Received: 19 November 2019; Accepted: 6 December 2019; Published: date

Table S1. Commercially available root canal sealers used in the studies included in this systematic review.

Type	Sealer	Manufacturer	In vitro	In vivo
**ZnO-eugenol**	PCS	Kerr, Romulus, USA	[24,27,29,34–36,44,45,61,62,66,90,88]	[95,107]
	PCS Extended Working Time	Kerr, Romulus, USA	[42,43]	[100]
	N2®	Indrag-Agsa, Losone, Switzerland	[48,58–60,65,80,81]	–
	Endofill	Produits Dentaires, Vevey Switzerland	[20,35,41,78]	[93]
	Canals	Showa Pharmaceutical Co., Tokyo, Japan	[58–60]	–
	Endométhasone	Septodont, Saint-Maur-des-Fossés, France	[48,65]	[97,106]
	Roth's Sealer	Roth International, Chicago, USA	[53,74]	–
	Grossman's sealer	Sultan Chemists, Englewood, USA	[31]	[103]
	Zinc Oxide-Eugenol (ZOE)	Produits Dentaires, Vevey Switzerland	[46,55]	–
	Tubli-Seal™	Kerr, Romulus, USA	[22,39]	–
	Tubli-Seal Xpress™	Kerr, Romulus, USA	[75]	–
	Cortisomol™	Pierre Rolland, Merignac, France	[47]	–
	Intrafill	Dentsply Ind. e Com. Ltda., Petrópolis, RJ, Brazil	–	[109]
**Resin (epoxy)**	AH Plus™	Dentsply DeTrey Gmbh, Konstanz, Germany	[17,20,22,23,30,32,33,37,38,43,44,47,48,50,52,53,55–57,61,64–66,68,69,71,74,76–78,82,84,85,89,91]	[92,94,104,109,110,112]
	AH 26®	Dentsply DeTrey Gmbh, Konstanz, Germany	[17,55,56,58–60,79–81,83,87,91]	[102,104]
	AH Plus Jet®	Dentsply DeTrey Gmbh, Konstanz, Germany	[25,29,36,39,40,49,54,75]	–
	Acroseal	Septodont, Saint-Maur-des-Fossés, France	[23,89]	–
	SimpliSeal®	Discuss Dental LLC, Calver City, USA	[20,73]	–
	TopSeal®	Dentsply DeTrey Gmbh, Konstanz, Germany	[34,38]	–
	Sealer Plus	MK Life, Porto Alegre, Brazil	[20]	–
	ThermaSeal®	Dentsply/Maillefer, Konstanz, Germany	[31]	–
	ThermaSeal® Plus	Dentsply/Maillefer, Konstanz, Germany	[53]	–

	Sicura Seal	Dentalica, Milano, Italy	[38]	–
**Resin (methacrylate)**	EndoREZ®	Ultradent, South Jordan, USA	[22,27,30,34,50,54,82,84]	[95,97,101,108]
	Epiphany®	Pentron, Wallingford, USA	[28,30–32,37,88,89]	[93,96,98,107,109]
	Epiphany® SE	Pentron, Wallingford, USA	[41,88]	–
	RealSeal™	SybronEndo, Orange, USA	[27,52,54,83]	[95]
	RealSeal SE™	SybronEndo, Orange, USA	[27,53]	–
	RealSeal XT	SybronEndo, Orange, USA	[25]	[105]
	MetaSEAL™	Parkell, Inc., Farmington, USA	[27,29]	–
	MetaSEAL™ Soft	Sun Medical, Tokyo, Japan	[39]	–
**Glass ionomer**	Ketac™ Endo	3M ESPE, St. Paul, USA	[48,65]	–
	Activ GP™	Brasseler, Savannah, USA	[83]	–
**Silicone**	GuttaFlow®	Roeko/Coltène/Whaledent, Langenau, Germany	[32,39,50,53,75,84]	–
	GuttaFlow®2	Roeko/Coltène/Whaledent, Langenau, Germany	[52,64,77]	[92]
	GuttaFlow® Bioseal	Roeko/Coltène/Whaledent, Langenau, Germany	[64]	[92]
	RoekoSeal	Roeko/Coltène/Whaledent, Langenau, Germany	[34,48,82]	[109]
	RoekoSeal Automix	Roeko/Coltène/Whaledent, Langenau, Germany	[30,33,90]	[104,110]
**Calcium hydroxide**	Sealapex™	Kerr, Romulus, USA	[22,26,31,47,53,80,81,90]	[113]
	Sealapex Xpress™	SybronEndo, Orange, USA	–	[105]
	Apexit®	Ivoclar Vivadent, Schaan, Liechtenstein	[48,50,65,84]	–
	Apexit® Plus	Ivoclar Vivadent, Schaan, Liechtenstein	[39]	–
	Sealer 26	Dentsply/Maillefer, Konstanz, Germany	[78]	[102,113]
**Bioactive**	MTA Fillapex®	Angelus, Londrina, Brazil	[23,26,39,40,41,51,57,61,64,66,71,73,76]	[103,112]
	MTA Angelus®	Angelus, Londrina, Brazil	[21,57,71]	–
	BioRoot™ RCS	Septodont, Saint-Maur-des-Fossés, France	[24,45,49,61–63,66,73]	–
	Endosequence BC™	Brasseler, Savannah, USA	[43,49,51,74–76]	–
	iRoot® SP	Innovative BioCeramix Inc., Vancouver, Canada	[26,40,68,72,86]	–

	iRoot® BP Plus	Innovative BioCeramix Inc., Vancouver, Canada	[46,67,70]	–
	iRoot® FS	Innovative BioCeramix Inc., Vancouver, Canada	[67,70]	–
	ProRoot® ES	Dentsply Tulsa Dental, Tulsa, USA	[74]	–
	Endoseal® MTA	Maruchi, Seoul, Korea	[63]	–
	MTA High plasticity	Angelus, Londrina, Brazil	[21]	–
	Apatite Root Sealer	Sankin Kogyo, Tokyo, Japan	[26,42]	[100]

Table S2. Results of risk of bias assessment of in vitro studies according to the guidelines for reporting of preclinical studies on dental materials by Faggion Jr. [18].

	Checklist item														
**Study**	**1**	**2a**	**2b**	**3**	**4**	**5**	**6**	**7**	**8**	**9**	**10**	**11**	**12**	**13**	**14**
Lee et al. [76]	Y	Y	Y	Y	Y	N	N	N	N	N	Y	N	N	Y	N
Jeanneau et al. [62]	Y	Y	Y	Y	Y	N	N	N	N	N	Y	N	N	Y	N
Giacomino et al. [74]	Y	Y	Y	N	Y	N	N	N	N	N	Y	N	N	N	N
Jung et al. [66]	Y	Y	Y	Y	N	N	N	N	N	N	Y	N	Y	N	N
Vouzara et al. [73]	Y	Y	Y	Y	Y	N	N	N	N	N	Y	N	N	N	N
Alsubait et al. [49]	Y	Y	Y	Y	N	N	N	N	N	N	Y	N	Y	Y	N
Jung et al. [61]	Y	Y	Y	Y	N	N	N	N	N	N	Y	N	Y	Y	N
Szczurko et al. [39]	Y	Y	Y	Y	Y	N	N	N	N	N	Y	N	Y	Y	N
Troiano et al. [38]	Y	Y	Y	Y	N	N	N	N	N	N	Y	Y	Y	N	N
Arun et al. [22]	Y	Y	Y	Y	Y	N	N	N	N	N	Y	Y	Y	N	N
Collado-González et al. [63]	Y	Y	Y	Y	N	N	N	N	N	N	Y	N	N	Y	N
Collado-González et al. [64]	Y	Y	Y	Y	N	N	N	N	N	N	Y	N	N	Y	N
Cintra et al. [21]	Y	Y	Y	Y	N	N	N	N	N	N	Y	N	N	Y	N
Zhu et al. [72]	Y	Y	Y	Y	N	N	N	N	N	N	Y	N	N	Y	N
Cintra et al. [20]	Y	Y	Y	Y	N	N	N	N	N	N	Y	N	N	Y	N
Lv et al. [70]	Y	Y	Y	Y	N	N	N	N	N	N	Y	N	N	Y	N
Victoria-Escandell et al. [57]	Y	Y	Y	Y	Y	N	N	N	N	N	Y	Y	N	Y	N
Suciu et al. [23]	Y	Y	Y	Y	N	N	N	N	N	N	Y	N	N	N	N
Camps et al. [45]	Y	Y	Y	Y	N	N	N	N	N	N	Y	N	N	Y	N
Dimitrova-Nakov et al. [24]	Y	Y	N	Y	N	N	N	N	N	N	Y	N	N	N	N
Konjhodzic-Prcic et al. [50]	Y	Y	Y	Y	Y	N	N	N	N	N	N	Y	N	Y	N
Konjhodzic-Prcic et al. [84]	y	N	N	Y	N	N	N	N	N	N	N	Y	N	N	N
Zhou et al. [51]	Y	Y	Y	Y	Y	N	N	N	N	N	Y	N	Y	Y	N
Silva et al. [77]	Y	Y	Y	Y	N	N	N	N	N	N	Y	N	N	Y	N
Parirokh et al. [56]	Y	Y	Y	Y	N	N	N	N	N	N	Y	N	N	Y	N
Jiang et al. [67]	Y	Y	Y	Y	Y	N	N	N	N	N	N	N	N	Y	N
Cotti et al. [25]	Y	Y	Y	Y	Y	N	N	N	N	N	Y	N	N	N	N
Chang et al. [26]	Y	Y	Y	Y	N	N	N	N	N	N	Y	N	N	Y	N
Mandal et al. [52]	Y	Y	Y	Y	Y	N	N	N	N	N	Y	N	N	N	N
Camargo et al. [82]	Y	Y	Y	Y	Y	N	N	N	N	N	Y	N	N	Y	N
Güven et al. [40]	Y	Y	Y	Y	N	N	N	N	N	N	N	N	N	N	N
Kim et al. [85]	Y	Y	Y	Y	N	N	N	N	N	N	Y	N	N	N	N
De-Deus et al. [46]	Y	Y	Y	Y	N	N	Y	N	N	N	Y	N	Y	Y	N
Bin et al. [71]	Y	Y	Y	Y	Y	N	N	N	N	N	Y	N	N	N	N
Scelza et al. [53]	Y	Y	Y	Y	Y	N	N	N	N	N	Y	N	N	Y	N
Salles et al. [41]	Y	Y	Y	Y	N	N	N	N	N	N	Y	N	N	N	N
Landuyt et al. [54]	Y	Y	Y	Y	Y	N	N	N	N	N	Y	Y	N	Y	N
Shon et al. [42]	Y	Y	Y	Y	N	N	N	N	N	N	Y	N	N	N	N

Mukhtar-Fayyad [86]	Y	Y	Y	Y	Y	N	N	N	N	N	Y	N	N	N	N
Zoufan et al. [75]	Y	Y	Y	Y	Y	N	N	N	N	N	Y	N	N	N	N
Loushine et al. [43]	Y	Y	Y	Y	Y	Y	N	N	N	N	Y	Y	Y	N	N
Brackett et al. [36]	Y	Y	Y	Y	Y	N	N	N	N	N	Y	N	N	N	N
Yu et al. [87]	Y	Y	Y	Y	N	N	N	N	N	N	Y	N	N	N	N
Zhang et al. [68]	Y	Y	Y	Y	N	N	N	N	N	N	Y	N	N	N	N
Huang et al. [58]	Y	Y	Y	Y	Y	N	N	N	N	N	Y	N	N	Y	N
Bryan et al. [44]	Y	Y	Y	Y	Y	Y	N	N	N	N	Y	N	N	N	N
Ames et al. [27]	Y	Y	Y	Y	Y	N	N	N	N	N	Y	Y	N	Y	N
Donadio et al. [83]	Y	Y	Y	Y	Y	N	N	N	N	N	Y	N	N	N	N
Gambarini et al. [88]	Y	Y	Y	Y	Y	N	N	N	N	N	Y	Y	N	N	N
Camargo et al. [89]	Y	Y	Y	Y	Y	N	N	N	N	N	Y	N	N	Y	N
Huang et al. [59]	Y	Y	Y	Y	N	N	N	N	N	N	Y	N	Y	Y	N
Heitman et al. [28]	Y	Y	Y	Y	Y	N	N	N	N	N	Y	N	Y	N	N
Valois and Azevedo [78]	Y	Y	Y	Y	Y	N	N	N	N	N	Y	N	N	N	N
Pinna et al. [29]	Y	Y	Y	Y	Y	Y	N	N	N	N	Y	Y	N	N	N
Huang et al. [60]	Y	Y	Y	Y	N	N	N	N	N	N	Y	N	N	Y	N
Lodienė et al. [30]	Y	Y	Y	Y	Y	N	N	N	N	N	Y	N	N	N	N
Lee et al. [80]	Y	Y	Y	Y	N	N	N	N	N	N	Y	N	N	Y	N
Lee et al. [79]	Y	Y	Y	N	N	N	N	N	N	N	Y	N	N	Y	N
Lee et al. [81]	Y	Y	Y	Y	N	N	N	N	N	N	Y	N	N	N	N
Merdad et al. [37]	Y	Y	Y	Y	Y	N	N	N	N	N	Y	N	N	Y	N
Key et al. [31]	Y	Y	Y	Y	Y	N	N	N	N	N	Y	N	N	N	N
Bouillaguet et al. [32]	Y	Y	Y	Y	Y	N	N	N	N	N	Y	N	N	N	N
Miletic et al. [33]	Y	Y	Y	Y	Y	N	N	N	N	N	Y	N	N	N	N
Al-Awadhi et al. [90]	Y	Y	Y	Y	Y	N	N	N	N	N	Y	Y	N	N	N
Bouillaguet et al. [34]	Y	Y	Y	Y	Y	N	N	N	N	N	Y	N	N	N	N
Camps and About [47]	Y	Y	Y	Y	Y	N	N	N	N	N	Y	Y	N	N	N
Mendes et al. [35]	Y	Y	Y	Y	N	N	N	N	N	N	Y	N	N	N	N
Schwarze et al. [48]	Y	Y	Y	Y	N	N	N	N	N	N	Y	N	N	N	N
Huang et al. [91]	Y	Y	Y	Y	Y	N	N	N	N	N	Y	Y	N	Y	N
Schwarze et al. [65]	Y	Y	Y	Y	N	N	N	N	N	N	Y	N	N	N	N
Azar et al. [55]	Y	Y	Y	Y	Y	N	N	N	N	N	Y	Y	N	N	N
Huang et al. [17]	Y	Y	Y	Y	Y	N	N	N	N	N	Y	Y	N	Y	N
Schweikl and Schmalz [69]	Y	Y	Y	N	Y	N	N	N	N	N	N	Y	N	N	N

Abbreviations: N, No; Y, Yes. Checklist items: 1 – Structured abstract; 2a – Scientific background and rationale; 2b – Objectives and/or hypotheses; 3 – Intervention of each group; 4 – Outcomes definition; 5 – Sample size determination; 6 – Allocation sequence generation; 7 – Allocation concealment mechanism; 8 – Implementation; 9 – Blinding; 10 – Statistical methods; 11 – Outcomes and estimation; 12 – Limitations; 13 – Funding information; 14 – Protocol (if available)

Table S3. Results of risk of bias assessment of in vivo studies according to the Systematic Review Centre for Laboratory Animal Experimentation (SYRCLE)'s risk of bias tool [19].

Study	Checklist item									
	**1**	**2**	**3**	**4**	**5**	**6**	**7**	**8**	**9**	**10**
Santos et al. [92]	U	Y	N	N	N	N	Y	Y	Y	N
Assmann et al. [112]	Y	Y	N	N	N	Y	Y	Y	Y	Y
Silva et al. [105]	N	N	N	N	N	N	Y	Y	Y	N
Zmener et al. [103]	N	Y	N	N	N	N	Y	Y	Y	N
Suzuki et al. [106]	Y	Y	N	N	N	N	Y	Y	Y	N
Garcia et al. [93]	U	Y	N	N	N	N	N	Y	Y	N
Oliveira et al. [94]	Y	Y	N	N	N	N	Y	Y	Y	Y
Brasil et al. [107]	Y	Y	N	N	N	N	Y	N	Y	N
Zmener et al. [95]	N	Y	N	N	N	N	Y	U	Y	N [1]
Suzuki et al. [108]	Y	Y	N	N	N	N	Y	Y	Y	N
Tanomaru-Filho et al. [109]	U	Y	N	N	N	N	Y	Y	Y	N
Derakhshan et al. [104]	Y	Y	N	N	N	N	Y	N	Y	N
Leonardo et al. [110]	U	Y	N	N	N	N	Y	Y	Y	N
Campos-Pinto et al. [96]	U	U	N	N	N	N	N	Y	Y	N
Zafalon et al. [97]	N	Y	N	N	N	N	N	Y	Y	N
Onay et al. [98]	U	Y	N	N	N	N	N	U	Y	N
Tanomaru-Filho et al. [113]	U	U	N	N	N	N	Y	Y	Y	N
Cintra et al. [111]	N	Y	N	N	N	N	Y	Y	Y	Y
Kim et al. [100]	N	N	N	N	N	N	N	Y	Y	Y
Zmener [101]	U	U	N	N	N	N	N	Y	Y	Y
Figueiredo et al. [102]	Y	Y	N	N	N	N	N	U	Y	N

[1] The preparation of sealer (EndoREZ with accelerator) was performed with slight modifications of the manufacturer's instructions. Also, one new animal was added to one of the groups (unspecified) to replace a drop-out from the original population (reasons were not specified).

Abbreviations: N, No; U, Unclear; Y, Yes. Checklist items*:* 1 – Allocation sequence generation; 2 – Baseline characteristics; 3 – Allocation concealment; 4 – Random housing; 5 – Caregiver and/or researcher blinding; 6 – Random outcome assessment; 7 – Outcome assessor blinding; 8 – Incomplete outcome data; 9 – Selective outcome reporting; 10 – Other sources of bias

© 2019 by the authors. Submitted for possible open access publication under the terms and conditions of the Creative Commons Attribution (CC BY) license (http://creativecommons.org/licenses/by/4.0/).
